# A multi-centre study on cervical cancer screening reveals population-related impact on cervical intraepithelial neoplasia rate

**DOI:** 10.3389/fcimb.2026.1920297

**Published:** 2026-09-28

**Authors:** Yun Zhao, Xinyu Wang, Rutie Yin, Mingzhu Li, Xiao Li, Qingli Li, Yu Wang, Jingran Li, Chao Zhao, Huiming Liao, Lihui Wei

**Affiliations:** 1 Department of Obstetrics and Gynaecology, Peking University People’s Hospital, Beijing, China; 2 Department of Gynaecology, The First Affiliated Hospital Zhejiang University School of Medicine, Zhejiang, China; 3 Department of Cancer Center, West China Second University Hospital, Sichuan University, Chengdu, Sichuan, China; 4 Department of Gynecologic Oncology, Women’s Hospital, School of Medicine Zhejiang University, Zhejiang, China; 5 Department of Obstetrics and Gynaecology, Beijing Shijitan Hospital, Capital Medical University, Beijing, China; 6 Department of Obstetrics and Gynecology, Shenzhen Fuyong People's Hospital, Guangdong, China

**Keywords:** age, cervical cancer screening, genotyping, HPV, repeat positivity

## Abstract

**Objective:**

To conduct a three-year cervical cancer screen in Zhejiang, Sichuan, Beijing, and Inner Mongolia and analyse clinical and population data to identify possible improvements to the screening protocol.

**Methods:**

A standard protocol including human papillomavirus (HPV) detection, cytology test, colposcopy, and histopathology inspection was adapted for the screening. Additional HPV genotyping was performed for participants in Sichuan, Beijing, and Inner Mongolia.

**Results:**

12,086 women were included in this study, and 178 cases of cervical intraepithelial neoplasia (CIN) grade 2 or worse (CIN2+) were identified. Detailed analyses on features of age, region, ethnicity, and origin showed a notable correlation between age strata and high-grade CIN rates. Moreover, apart from HPV16/18, other HPV types such as HPV33, HPV52, and HPV58 were found to be enriched in high-grade CIN cases, as was repeat positivity.

**Conclusions:**

Our results suggest that age, HPV16, and ThinPrep cytologic test (TCT) are prominent exploratory predictors for CIN2+ detection, and HPV33, 52, and 58 may pose higher risks than other non-HPV16/18 types. Future studies incorporating prospective effectiveness and health-economic evaluation are required to validate the clinical utility of these predictors.

## 1 Introduction

Cervical cancer is the fourth most common malignant tumour in women (Li et al., 2025), causing around 250,000 deaths annually in China (Cao et al., 2021). Fortunately, it is highly preventable as most cervical cancers are induced by human papillomavirus (HPV) infections (Crosbie et al., 2013). The common cervical cancer prevention approach implemented by China is screening with cytology inspection, HPV testing, and colposcopy-guided biopsy (Molina et al., 2024). However, due to complicated conditions across the country, screening protocols and coverage vary in different regions, and a more effective and adaptable protocol is required (Jinghe and Qiang, 2022).

Efforts were made to collect data worldwide to optimise resources and strategies for regional cervical cancer screening. Castellsagué et al. summarised HPV genotypes detected in cervical intraepithelial neoplasia (CIN) cases and reported differences in vaccination contribution between continents (Castellsagué et al., 2016), suggesting that screening strategies should align with local vaccination coverage. Some researchers focused on the burden of HPV-related diseases and emphasised that a higher burden is likely associated with limited screening resources in developing countries (Kombe Kombe et al., 2021). Perkins et al. pointed out that HPV detection can be used to replace cytology tests, especially in regions lacking screening resources (Perkins et al., 2023). Meanwhile, Wheeler et al. suggested that HPV genotyping may reduce unnecessary colposcopy examinations in cervical cancer screening (Wheeler et al., 2014). In China, although ThinPrep cytologic tests (TCTs) and HPV tests are the main screening methods for cervical cancer screening, similar but differing protocols were introduced by the national cervical cancer screening guidelines (Li et al., 2023) for adaptation according to local healthcare resources and requirements. In a more recent guideline for cervical cancer screening in China (Li et al., 2025), HPV testing was suggested for use as a primary test with TCTs rather than as a secondary test after TCT, and younger women, such as those aged 25 to 30, were advised to be included as part of the main target population.

In this study, HPV testing and TCTs were used as the primary tests for cervical cancer screening conducted in Zhejiang, Sichuan, Beijing, and Inner Mongolia. All high-risk HPV types were included in the detection assay, but only HPV16 and HPV18 were genotyped. Additional HPV genotyping was conducted for participants in Sichuan, Beijing, and Inner Mongolia, providing insights into regional HPV type distributions. Population features were combined with testing results to explore possible improvements to the current national protocol or potential adjustments for the regions involved.

## 2 Methodology

### 2.1 Study population and screening protocol

This research was conducted in 4 provinces (including a municipality): Sichuan, Zhejiang, Inner Mongolia and Beijing. Women between 20 and 65 years of age with a history of sexual activity were recruited for screening. The exclusion criteria were: (a) a previous diagnosis of cervical cancer; (b) current chemotherapy, radiotherapy, or both; (c) being pregnant or within 8 weeks postpartum; (d) a history of pelvic radiotherapy or chemotherapy, cervical resection within 12 months before the study, or total hysterectomy; and (e) a history of HPV vaccination. A 3-year screening and follow-up protocol, illustrated in **Figure 1**, was implemented. In brief, participants underwent routine testing (pooled detection of 18 high-risk HPV (hrHPV) types with separate identification of HPV16/18) and TCTs at the beginning of screening and were assigned to colposcopy assessment, next-year follow-up, or third-year follow-up according to the results. At the next-year follow-up, TCTs were performed, and participants with abnormal cytology were referred to colposcopy, while those with a TCT result negative for intraepithelial lesion or malignancy (NILM) were scheduled for another follow-up before the third year. At the third-year follow-up, HPV genotyping and TCTs were performed, and participants with a positive HPV result or abnormal cytology were referred to colposcopy. Participants who underwent colposcopy had histological samples taken for pathological diagnosis. Those diagnosed with CIN2 or worse (CIN2+) left the study and were treated accordingly, while the others continued to the next follow-up until the end of the study. Extended genotyping was available for all Sichuan participants, for participants enrolled during the last three screening rounds in Beijing and Inner Mongolia, and for none in Zhejiang. This was an operational, site- and round-dependent implementation rather than a random sample of the full cohort. Repeat positivity was defined as positive results at two consecutive available HPV tests. Because routine HPV testing was not performed in Years 1 and 2, and extended genotyping was not uniformly conducted at baseline, same-genotype continuity was not consistently demonstrable. Therefore, clearance, reinfection, new infection, and true persistence cannot be distinguished, and this measure must not be interpreted as guideline-defined persistent infection.

**Figure 1:**
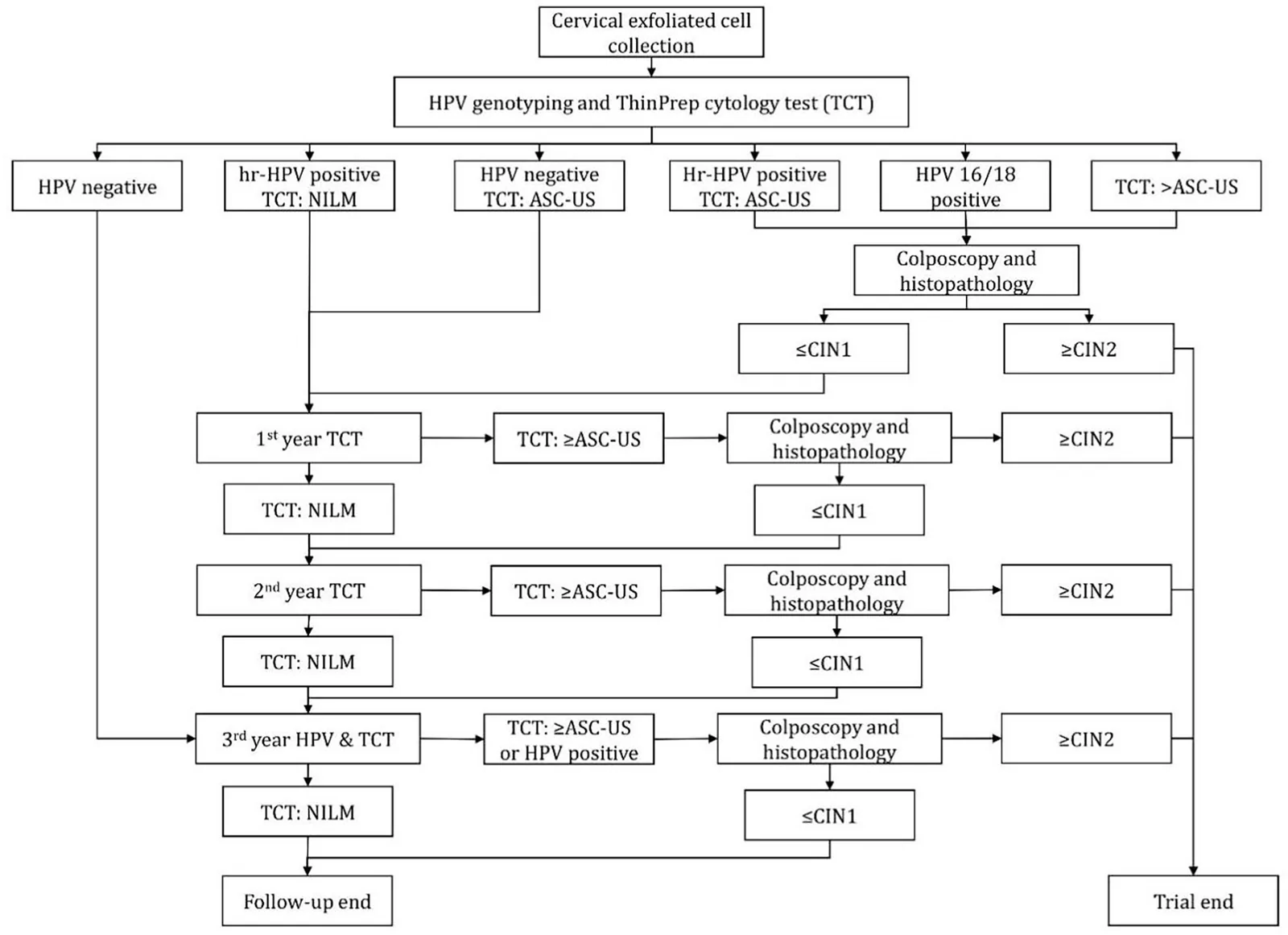
Process of cervical cancer screening.

### 2.2 Sample collection

Cervical exfoliated cells were collected by gynaecologists using collectors (Hologic Inc., Marlborough, MA, USA) and preserved in PreservCyt Solution (Hologic Inc., Marlborough, MA, USA) for TCT and HPV genotyping within 48 h.

### 2.3 HPV detection

DNA was extracted using DNA extraction kits (Yaneng BIOscience (Shenzhen) Co., Ltd., Shenzhen, Guangdong, China), and HPV DNA was routinely tested using HPV DNA detection and HPV16/18 genotyping kits (PCR-fluorescence probing; Yaneng BIOscience (Shenzhen) Co., Ltd., Shenzhen, Guangdong, China), which can detect 18 hrHPV types in a pooled assay (16, 18, 26, 31, 33, 35, 39, 45, 51, 52, 53, 56, 58, 59, 66, 68, 73, and 82) and separately genotype HPV16 and HPV18. For all extended type-specific genotyping, HPV DNA detection and genotyping kits (PCR-reverse dot hybridisation; Yaneng BIOscience (Shenzhen) Co., Ltd., Shenzhen, Guangdong, China) were used. The detection experiments were conducted strictly according to the manufacturer’s instructions.

### 2.4 Liquid-based cytology

Exfoliated cell cytology was examined by experienced pathologists according to the 2014 Bethesda System and characterised as follows: (a) negative for intraepithelial lesion or malignancy (NILM); (b) atypical squamous cells of undetermined significance (ASC-US); (c) atypical squamous cells, cannot exclude high-grade squamous intraepithelial lesion (HSIL) (ASC-H); (d) atypical glandular cells (AGC); (e) low-grade squamous intraepithelial lesion (LSIL); and (f) high-grade squamous intraepithelial lesion (HSIL).

### 2.5 Colposcopy and histopathology

Colposcopies were performed by gynaecologists, and biopsies were performed when visible lesions were found. The histopathological examinations were then performed to establish final diagnoses of <CIN1, cervical intraepithelial neoplasia (CIN) grades 1 to 3, and squamous cell carcinoma (SCC).

### 2.6 Statistical analysis and modelling

Statistical analyses were performed using Python 3.6 with the NumPy module. Modelling, including cross-validation and permutations, was performed using the scikit-learn module. Before being entered into the models, features were coded as unordered factors, except for age, which was coded numerically. The random forest (RF) models were constructed with default parameters and 100 trees. Permutations were performed 10 times to calculate the permutation importance of each feature. The shap package in Python was used to calculate Shapley additive explanations (SHAP) values (Lundberg and Lee, 2017) and perform further analyses and visualisation of these values. It should be noted that the full baseline dataset was used for model fitting and importance estimation; there was no independent test set. Therefore, RF/SHAP results were considered exploratory associations, not causal effects. For age adjustment, direct standardisation was used across five prespecified age strata (<30, 30–39, 40–49, 50–59, and ≥60 years). The standard population was the age distribution of all 12,086 participants. For each subgroup, the age-standardised CIN2+ rate was calculated as Σ(w_i_ × r_i_), where w_i_ is the proportion of the full cohort in age stratum i and r_i_ is the subgroup-specific CIN2+ rate in that stratum. To evaluate differences in CIN2+ rates between subgroups, a global Pearson chi-square test was used to compare the four regional Han groups. Prespecified two-sided Fisher’s exact tests were applied for pairwise comparisons where expected counts were low. All statistical tests were two-sided, and *P* < 0.05 was considered statistically significant.

## 3 Results

### 3.1 Cervical screening profile

Overall, 12,086 women aged 20 to 65 years, with an average age of 44.83 ± 8.93 years, from four different provinces or municipalities were involved in this study (**Table 1**). The majority of participants were Han; ethnic minority groups with more than 50 participants included Yi, Mongol, Hui, and Man, while the remaining minorities were grouped as “other” and were not listed in detail. According to information on the participants’ personal IDs, more women were born in rural areas than in urban areas. At first enrolment, the positive rate for HPV detection was 14.81%, the abnormal rate for TCTs was 7.84%, and 93 patients were diagnosed with CIN2+ and referred for further treatment (**Table 2**). Another 85 patients were diagnosed with high-grade CIN or SCC during follow-up screening and left the study for treatment. The overall cumulative three-year CIN2+ rate of the cohort was 1.47% (178/12,086), while the baseline rate was 0.77% (93/12,086). CIN2+ rates among participants who underwent colposcopy at specific time points were 16.03% (93/580) at baseline, 17.36% (21/121) in Year 1, 7.27% (12/165) in Year 2, and 4.62% (52/1,126) in Year 3, respectively. HPV was detected only at enrolment and in the third year according to the screening protocol, but additional HPV genotyping tests were performed for participants in Sichuan, Beijing, and Inner Mongolia, and the results are presented in the following sections.

**Table 1:** Participant population profile.

Category	Subgroup	Counts	Proportion (%)
**Total**		12086	100.00%
**Age**	(-, 30)	536	4.43%
	[30, 40)	3051	25.24%
	[40, 50)	4535	37.52%
	[50, 60)	3402	28.15%
	[60, -)	562	4.65%
**Ethnic**	Han	10897	90.16%
	Yi	736	6.09%
	Mongol	293	2.42%
	Hui	73	0.60%
	Man	64	0.53%
	Other	23	0.19%
**Region**	Zhejiang	3028	25.05%
	InnerMongolia	2774	22.95%
	Beijing	3103	25.67%
	Sichuan	3181	26.32%
**Origin**	Urban	4227	34.97%
	Rural	7859	65.03%

**Table 2:** Overview of cervical cancer screening results.

	Variable	Baseline	Year 1	Year 2	Year 3
HPV	HPV-	10296	Not performed	Not performed	8155
	HPV16	224	Not performed	Not performed	88
	HPV18	108	Not performed	Not performed	40
	HPV16/18	7	Not performed	Not performed	0
	hrHPV	1451	Not performed	Not performed	911
	Total	12086 (100.00%)	/	/	9194 (100.00%)
TCT	NILM	11138	1694	1725	8727
	ASC-US	687	106	187	380
	ASC-H	76	10	9	24
	AGC	29	5	3	8
	LSIL	112	29	19	46
	HSIL	44	5	3	5
	Total TCT tested	12086 (100.00%)	1849 (100.00%)	1946 (100.00%)	9190 (100.00%)
Histopathology	<CIN1	373	72	129	783
	CIN1	114	28	24	291
	CIN2	40	9	9	37
	CIN3	52	12	3	14
	SCC	1	0	0	1
	CIN2+ subtotal	93	21	12	52
	Total	580 (100.00%)	121 (100.00%)	165 (100.00%)	1126 (100.00%)

### 3.2 Population feature analysis

Rates of CIN2+ were investigated in different population groups. Only data from the first screen were used in this analysis, as follow-up was performed in a subgroup of the same population. Population features including age, origin (urban/rural born), ethnicity, and location (province or municipality) of testing, and clinical features derived from HPV test and TCT results were considered as features potentially associated with CIN2+ rates. As only 93 cases of CIN2+ were identified at the first screen, this number of positive cases was insufficient to perform robust statistical analyses on these eight features. Therefore, classification random forest (RF) models were used to assist the analysis by estimating the importance of these features.

As the main aim was to estimate feature importance rather than to train a general model, the full baseline dataset was used for model fitting and importance estimation; there was no independent test set. This design creates risks of overfitting and optimistic importance estimates. Therefore, RF/SHAP results were considered exploratory associations, not causal effects. A 10-fold cross-validation resulted in an average area under the receiver operating characteristic curve (AUC) of 0.884 with a standard deviation of 0.078, indicating that the model was able to capture relations between these features and the CIN2+ results without excessive overfitting. Feature importance was assessed using permutation-induced changes in prediction accuracy and SHAP values. Feature importance results are shown in **Figure 2**, where permutation importance, expressed as decreases in accuracy, is presented in **Figure 2a**; SHAP importance, expressed as the mean absolute SHAP value, is presented in **Figure 2b**; and the corresponding distributions of SHAP values across features are presented in **Figure 2c**. Although the order of importance varied between estimation methods, it is clear that TCT results, age, and HPV16 infections are strong exploratory indicators of CIN2+ diagnoses. Notably, the difference between the SHAP values for age (0.12) and HPV16 (0.13) was negligible, indicating that both age and HPV16 serve as important predictors with comparable predictive contributions. Meanwhile, participants’ origin, ethnicity, and HPV18 infection had lower estimated importance. It is worth noting that reliable estimates of the importance of HPV infection-related features would be largely affected by the number of infections with the relevant HPV type, since rare cases could not provide enough information to establish relationships within the models. Therefore, infections with other non-16/18 hrHPV types, including some uncommon types, were grouped into a single feature. Among the population-related features, age appeared to be the most important, followed by region and origin, while ethnicity was suggested by the models to be the least important. The SHAP value distribution of the age feature (**Figure 2c**) exhibited a more complicated nonlinear association with CIN2+ than those of the TCT and HPV-related features, requiring more detailed investigation.

**Figure 2:**
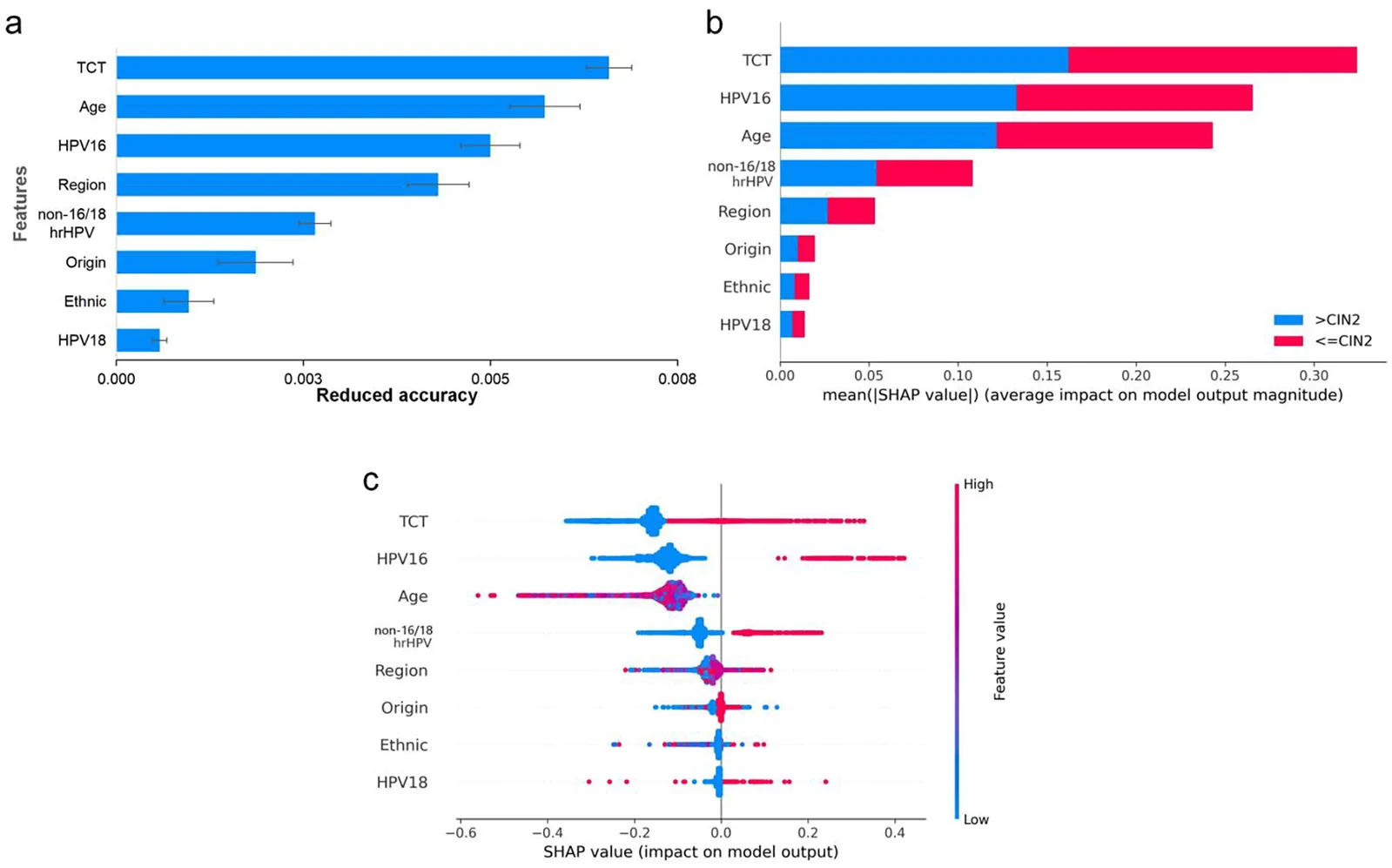
Feature importance estimated by permutation and SHAP. **(a)** Permutation importance calculated as the mean reduction in accuracy, with error bars representing the standard deviations over 10 repeats. **(b)** SHAP importance as the mean absolute SHAP value for different classes, and **(c)** the corresponding distributions of SHAP values across different features. “Non-16/18 hrHPV” represents infection with high-risk HPV types other than HPV16/18. The “origin” feature represents the urbanisation level of participants’ families at birth.

To verify the feature-importance estimates produced by the models, CIN2+ rates were examined in subgroups separated by region, origin, and ethnicity. Only subgroups with populations over 200 were investigated in detail, as large errors may be introduced given that the overall CIN2+ rate was merely 1.47% (see **Supplementary Table 1** for detailed data). To explore regional differences in CIN2+ rates, a comparison of the crude and age-adjusted rates among Han women in different regions (**Table 3**) showed variation between regions, with the lowest CIN2+ rate, 0.43%, in Zhejiang and the highest, 0.93%, in Inner Mongolia. However, a global Pearson chi-square test of these four regional Han groups showed no statistically significant difference (χ²(3) = 6.09, *P* = 0.107). Similarly, pairwise comparisons using Fisher’s exact tests revealed no significant differences in CIN2+ rates between subgroups based on origin or ethnicity. Thus, while some numerical variations in crude rates were observed across subgroups, these differences did not reach statistical significance in this cohort.

**Table 3:** CIN2+ rates of different population subgroups.

Subgroup			CIN2+/N	CIN2+ rate	Directly standardised CIN2+ rate	P value
Region	Ethnicity	Origin				
Zhejiang	Han	All	13/3028	0.43%	0.43%	0.107^a^
Beijing	Han	All	28/3103	0.90%	0.90%	
Inner Mongolia	Han	All	25/2774	0.92%	0.93%	
Sichuan	Han	All	23/3181	0.72%	0.74%	
Beijing	Han	Rural	17/1808	0.94%	0.93%	0.805^b^
Beijing	Han	Urban	11/1295	0.72%	0.81%	
Sichuan	Han	Rural	9/1608	0.56%	0.60%	0.088^c^
Sichuan	Han	Urban	14/1573	1.28%	1.06%	
Sichuan	Yi	Rural	13/1215	1.07%	0.98%	0.176^d^
Inner Mongolia	Han	Rural	16/1770	0.90%	0.92%	0.494^e^
Inner Mongolia	Mongol	Rural	17/1405	1.21%	1.13%	

N, number of participants in each subgroup; CIN2+, cervical intraepithelial neoplasia grade 2 or worse.

Directly standardised rate = Σ(age-specific subgroup rate × corresponding full-cohort age weight), using five age strata.

P values were calculated based on the counts in **Supplementary Table 1**.

^a^Global Pearson chi-square test comparing CIN2+ rates across the four regional Han groups (Zhejiang, Beijing, Inner Mongolia, Sichuan).

^b^Two-sided Fisher’s exact test comparing Beijing Rural vs. Beijing Urban Han.

^c^Two-sided Fisher’s exact test comparing Sichuan Rural vs. Sichuan Urban Han.

^d^Two-sided Fisher’s exact test comparing Sichuan Rural Yi vs. Sichuan Rural Han.

^e^Two-sided Fisher’s exact test comparing Inner Mongolia Rural Mongol vs. Inner Mongolia Rural Han.

Compared with other features, age can be investigated in more subgroups. Because participant numbers for those aged under 30 and over 60 were lower, causing considerable variability in the results, the analysis focused on women aged 30 to 60. In subgroups with sufficient sample sizes, such as urban Han from Zhejiang, rural Han from Inner Mongolia and Sichuan, urban and rural Han from Beijing, and Yi from Sichuan, CIN2+ rates in the 30–39 age group were significantly higher than those in the 40–49 age group (*P* = 0.0434) and 50–59 age group (*P* = 0.009). A more straightforward presentation of CIN2+ rates of different age strata was illustrated in **Figure 3a**. As HPV infection is a main cause of CIN2+, HPV infection rates in these subgroups were calculated and presented in **Figure 3b**. However, the expected pattern, similar to that of the CIN2+ rate, was not observed (all *P* > 0.05), but another pattern of infection rates increasing with age was observed in most subgroups. It is worth noting that general high-risk HPV infection rates were used here instead of type-specific infection rates because of insufficient data. It can be speculated that CIN2+ is more strongly associated with infection rates of certain HPV types than with general high-risk HPV infection rates, given differences in pathogenicity between HPV types.

**Figure 3:**
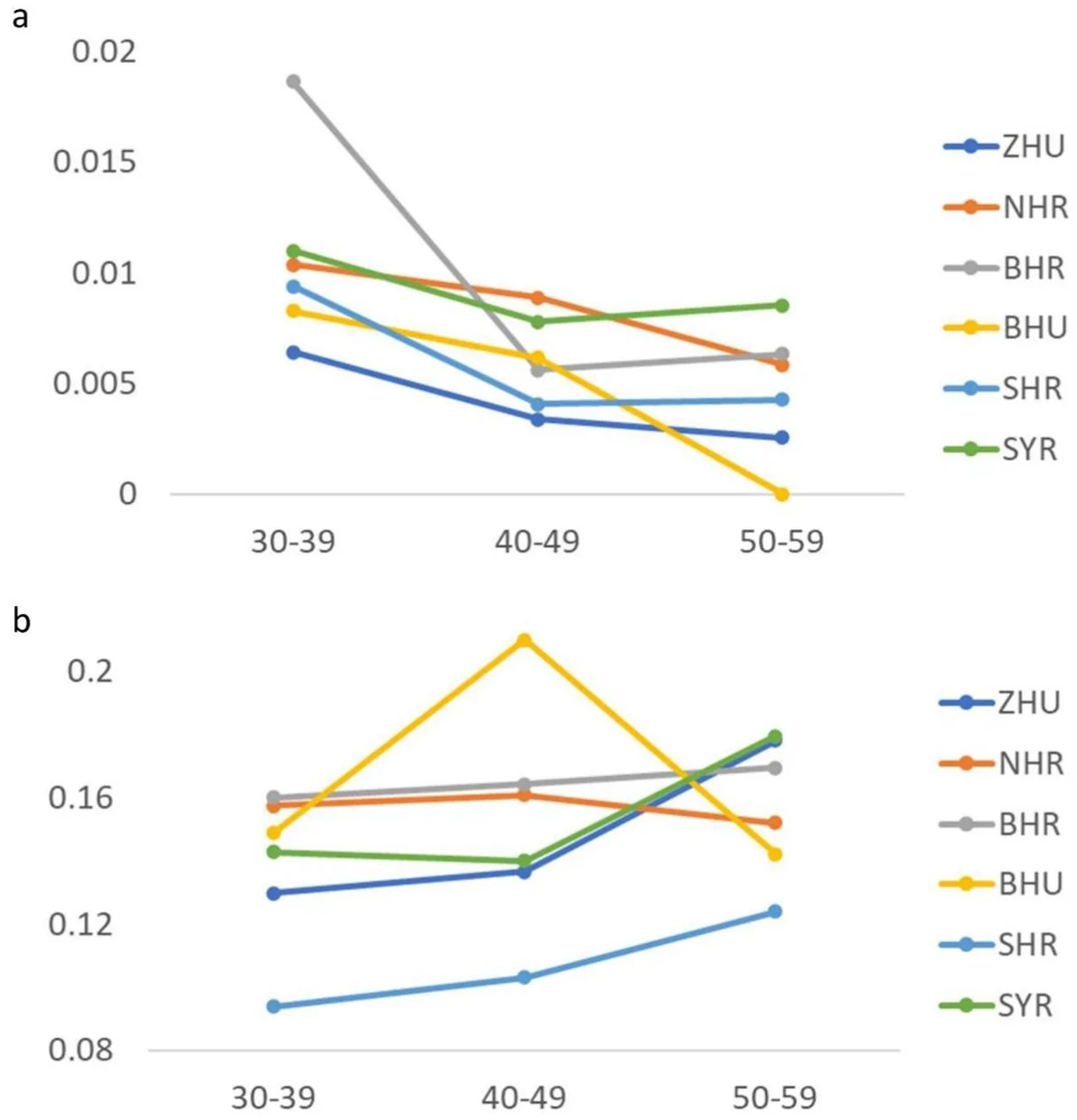
CIN2+ rates and HPV infection rates in different age strata in different population subgroups. **(a)** CIN2+ rates showed a significant decreasing trend with age (30–39 vs. 40–49: *P* = 0.0434; 30–39 vs. 50–59: *P* = 0.009). **(b)** HPV infection rates exhibited no significant differences across age strata (all *P* > 0.05). Comparisons were performed using Student’s *t*-test.” ZHU: urban Han women in Zhejiang; NHR: rural Han women in Inner Mongolia; BHR: rural Han women in Beijing; BHU: urban Han women in Beijing; SHR: rural Han women in Sichuan; SYR: rural Yi women in Sichuan.

### 3.3 High-grade lesions found in the follow-up period

There were 85 patients diagnosed with CIN2+ during the follow-up period. Additional annual HPV tests in some patients made it possible to track HPV infection history on a yearly basis. A total of 52 patients with CIN2+ were diagnosed in the last year; of these, 20, accounting for 23.53% of all CIN2+ cases identified during follow-up and 38.46% of those identified in the last year, had normal TCT results and were HPV-negative at baseline and therefore were not revisited in Years 1 and 2. Detailed analyses of these cases showed a significant overrepresentation of patients aged 50–59 (**Figure 4a**) relative to the age distributions of all CIN2+ cases (*P* = 0.015) and all participants (*P* = 0.001). Meanwhile, the HPV type distribution of these 20 cases showed no significant difference (*P* = 0.391) compared to that of all CIN2+ cases (**Figure 4b**). Rather than being solely age-related, this concentration in the 50–59 age group may reflect the absence of routine HPV retesting in Years 1 and 2 and the relatively high false-negative rate of cytological screening in this age bracket, which delayed lesion ascertainment until Year 3, thereby confounding the biological effects of age with the surveillance bias introduced by the screening interval design. Therefore, this pattern cannot be attributed solely to age factors.

**Figure 4:**
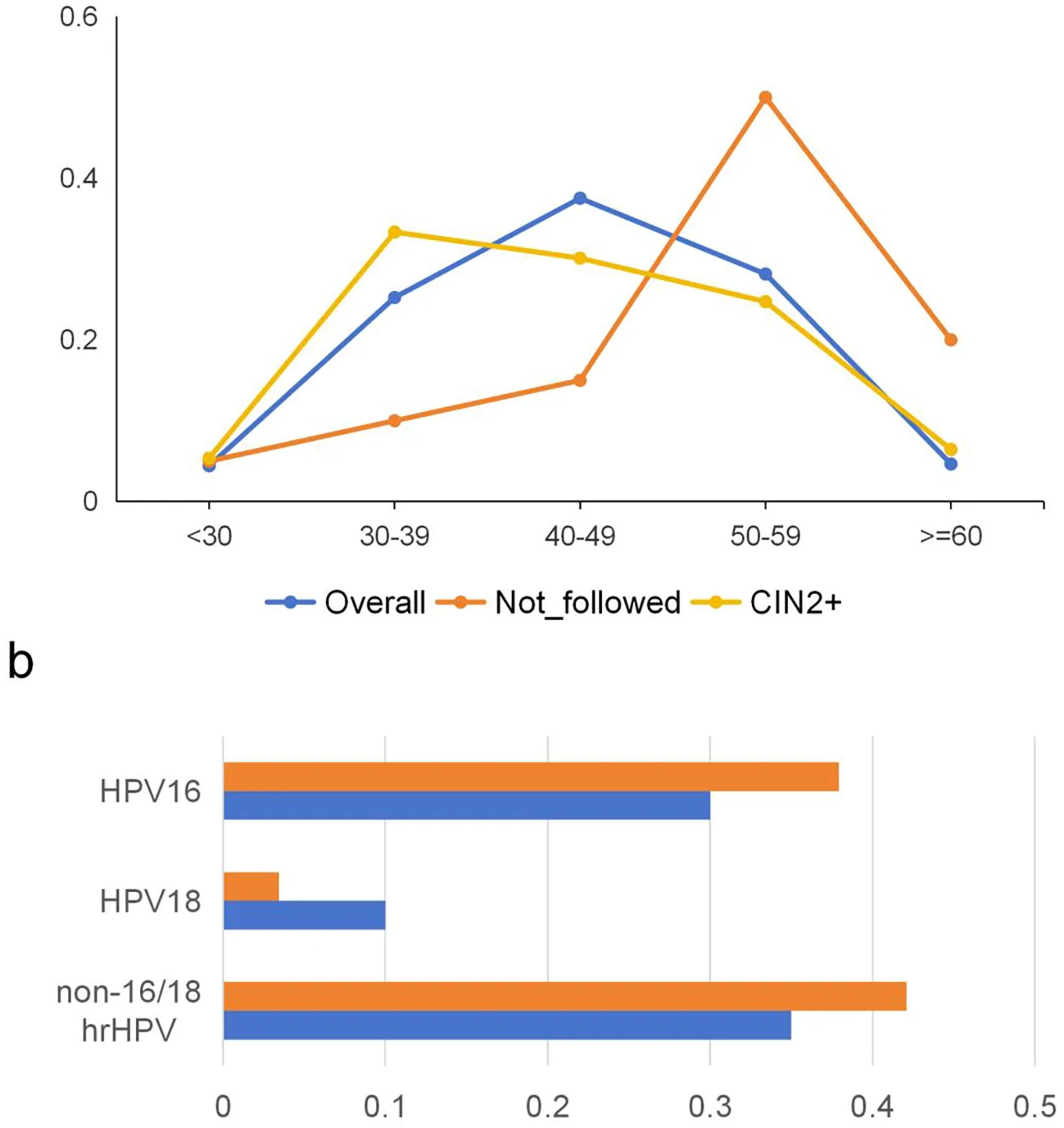
Age and HPV type distribution of the high-grade lesion cases not followed annually. **(a)** The age distribution of the high-grade lesion (CIN2+) cases not followed annually was compared to the age distributions of all CIN2+ cases and all participants. The differences in distribution between the group not followed annually and each of the other two groups were significant: *P* = 0.015 for all CIN2+ cases and *P* = 0.001 for all participants. **(b)** The HPV type distribution of the CIN2+ cases not followed annually was compared to that of all CIN2+ cases. The difference between these two distributions was not significant (*P* = 0.391). Non-16/18 hrHPV: high-risk HPV types other than HPV16 and HPV18.

Besides, 14 of the 52 CIN2+ cases identified were referred to colposcopy based on HPV infection without abnormal TCT results, indicating that using abnormal TCT results as the sole criterion for further colposcopy assessment may miss CIN2+ cases. Therefore, 42 CIN2+ cases diagnosed during the follow-up period, with a history of HPV infection and previously normal cytology, were further investigated. Amongst these patients, excluding those lacking additional HPV test data, only 3 (7.14%) had no evidence of repeat non-16/18 hrHPV positivity or previous HPV16/18 infection, while 20 (47.62%) had repeat non-16/18 hrHPV positivity and 12 (28.57%) had a history of HPV16/18 infection, suggesting that repeat non-16/18 hrHPV positivity may also be an important indicator of high-grade CIN. In the above description, repeat hrHPV positivity was defined as positive results at two consecutive available HPV tests. These results suggest that attention should be paid to repeat non-16/18 hrHPV positivity in addition to HPV16/18 infections in cervical cancer screening.

### 3.4 High-risk HPV genotyping

The results presented in the previous sections indicated that high-risk HPV types other than HPV16/18 infected a notable proportion of patients and that infection with these types was a less important feature than region, HPV16 infection, and age. The extended type-specific genotyping allows closer investigation of these infections. In total, extended genotyping data were available for 1,039/1,753 histopathologically evaluated participants (59.27%; 8.60% of the full cohort), including 983/1,575 with CIN1 or less and 56/178 with CIN2+; the distributions of different HPV types among the CIN1-, CIN2+, and CIN2+ with repeat positivity groups are shown in **Figure 5**. The proportions of certain types, such as HPV16, HPV33, HPV52, and HPV58, increased in CIN2+ compared with CIN1-, while those of others, such as HPV51, HPV53, HPV59, and HPV68, decreased. Differences in proportions between CIN2+ cases with repeat positivity and CIN2+ cases overall were also observed, but only 20 cases of repeat positivity were captured, including cases with multiple HPV infections, indicating that more data are required to draw reliable conclusions. These changes in infection proportions suggested differences in oncogenicity among HPV types, which may be used to inform more precise screening protocols based on HPV type.

**Figure 5:**
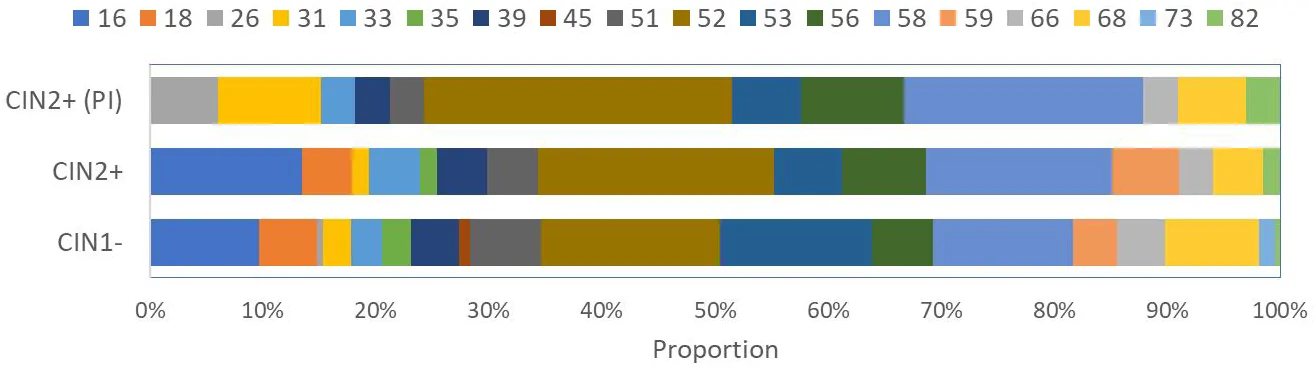
High-risk HPV type distributions among patients in the CIN2+ with repeat positivity, CIN2+, and CIN1- groups. HPV types and their corresponding colours are listed at the top. Repeat positivity was defined as positive results at two consecutive available HPV tests. CIN1-: not worse than CIN1; CIN2+: CIN2 or worse.

## 4 Discussion

### 4.1 Model-assisted feature importance analysis

RF models were implemented to identify crucial features from the screening data and produced results that were verified by analyses of subgroups with sufficient sample sizes. As the full baseline dataset was used for model fitting without an independent test set, these results are exploratory associations rather than causal effects. The importance of feature selection has been recognised in machine learning (Parikh and Zitnick, 2010) and data mining (Visalakshi and Radha, 2014), making it a standalone research topic (Kaur et al., 2021) in machine learning. However, the application of these techniques has only recently extended to medical practice alongside artificial intelligence and has mostly involved visual recognition (Abdualgalil and Abraham, 2020). We found these techniques are particularly useful in analysing complex data with multiple categorical variables with different numbers of categories, together with age as a numerical variable, which makes them powerful tools for analysing big data, such as population data and epidemiologic data, for assisting decision making or protocol adjusting.

SHAP values and permutation importance were both adopted in this study to provide perspectives with and without feature interactions. While SHAP values are obtained by considering influences between features (Lundberg and Lee, 2017), permutation importance is calculated by shuffling values of the target feature regardless of its relation to other features. Applying both methods may outline dependent and independent importance of the features, and when differences in importance are reported by the different methods, relationships between the relevant features can be analysed further. Besides, compared with permutation importance, SHAP values may provide further information on feature influence for individual samples, improving model transparency.

### 4.2 Potential application of the age factor in cancer screening

In this study, cervical cancer risk was associated with population features, such as region, ethnicity, urbanisation level at birth, and age, along with HPV infections and TCT results. Among these population features, some, such as region and ethnicity, displayed notable but not quantifiable associations with histopathology results, while age showed similar associations repeatedly across regions and ethnicities in this study. Furthermore, CIN2+ cases identified in participants not followed annually showed a clear bias toward older participants. However, this pattern may reflect surveillance bias and lower TCT sensitivity in older women, rather than a purely biological age effect. The biological effect of age and the surveillance-bias effect are confounded and cannot be separated in the present design. HPV prevalence has been reported for a number of regions across China by age stratum (Yan et al., 2021; Liu et al., 2022; Liu et al., 2023), but age has rarely been analysed separately from other features or in depth. CIN2+ rates and HPV infection rates for certain populations were reported here by age stratum and showed similar intergroup profiles, but the relevant case numbers were small, limiting the reliability of these results. In addition, other researchers have reported similar HPV infection profiles but different CIN2+ profiles in other populations (Castellsagué et al., 2016; Song et al., 2020). Meanwhile, conflicting results suggesting that the importance of age is unclear have also been reported (Carozzi et al., 2014). A lack of attention to older women was noted by some researchers, but the relevant characteristics were not compared with those of other age groups to establish age-specific screening protocols (Chen et al., 2025). The main cause of the inconsistency between these profiles has not been fully identified, so further investigation is required.

### 4.3 Test frequency and HPV infection history

According to local guidelines (Branch of Cancer Prevention and Control CPMA et al., 2023), persistent HPV infection was defined as testing positive for high-risk HPV in two consecutive tests at an interval of 6 to 12 months. This required a corresponding testing or screening frequency, consistent with the guideline requirements for those infected with HPV. The results of this study demonstrated a notable association between high-grade lesions and a history of high-risk HPV infection, highlighting the importance of considering repeat hrHPV positivity in screening. On the other hand, CIN2+ cases identified during the Year 3 follow-up accounted for 39.41% of the total 178 CIN2+ cases. Specifically, 20 cases (38.46% of Year 3 cases, including one cancer) were identified in women not included in annual follow-up. These 20 protocol-delayed cases, concentrated at ages 50–59, are likely attributable to surveillance bias and lower TCT sensitivity rather than age biology alone. These observations identify a surveillance limitation in the current protocol. However, earlier studies indicated that screening every 3–5 years in the general population was more economically efficient, as the benefits of shortening the screening interval to 1 year were lower than the associated increase in costs (Miller, 2003; Lees et al., 2016). Since this study did not randomise screening intervals, compare clinical strategies, or collect cost data, it cannot establish that shorter population-wide intervals are effective or cost-effective. Recommendations for screening optimisation require prospective effectiveness and health-economic evaluation. Besides, our results demonstrated insufficient willingness to undergo annual screening tests, indicating that more education and promotion regarding cervical cancer and HPV infection were required (Wang et al., 2023).

### 4.4 HPV genotyping for additional high-risk HPV types

Our results suggested that HPV genotype was another important factor in cervical cancer risk estimation. Different cancer risks associated with HPV types other than HPV16/18 have been reported in many studies (Carozzi et al., 2014; Li et al., 2024), but guidelines for these specific HPV types have not yet been published in China. This study suggests that HPV33, 52, and 58 may be associated with a higher cervical cancer risk than other non-HPV16/18 high-risk types, and similar results have been reported by other researchers (Yan et al., 2021; Song et al., 2022; Liu et al., 2023). Due to limited HPV genotyping data, only the most common high-risk types were mentioned in the results presented here, but other less common types should not be overlooked. For example, Chen et al. described the pathogenicity of HPV82 and its association with viral load (Chen et al., 2025), while the pathogenicity of HPV45 has been reported in studies conducted in other countries (Chen et al., 2014; Koestler et al., 2025). In this study, although HPV45 and HPV82 were detected in only a small number of cases, HPV82 was detected more frequently in CIN2+ cases and CIN2+ cases with repeat positivity, which agrees with the findings mentioned above. Moreover, an understanding of local HPV type distribution may enable in-depth studies of infections with multiple HPV types (Salazar et al., 2015; Kim et al., 2021) and more efficient vaccination (Jiang et al., 2019). Routine implementation of extended genotyping requires confirmation in uniformly tested populations to fully validate its clinical utility and cost-effectiveness.

### 4.5 Limitations and future requirements

This study has several limitations. First, extended HPV genotyping was not uniformly performed for all participants. This site- and screening-round dependence can introduce selection bias and prevent population-wide inference. Second, routine HPV retesting was not conducted in Years 1 and 2 of the screening protocol, limiting the ability to assess type-specific persistence of HPV infections. Third, data on confounding factors such as smoking and sexual behaviour were not available, which may influence the interpretation of our findings. Fourth, the number of CIN2+ outcomes was relatively sparse, potentially reducing the statistical power of our analyses. Fifth, we did not use an independent test set for the random forest (RF) model, which may affect the generalisability of our feature importance results. Sixth, ethnic subgroups were small, limiting the ability to draw conclusions about ethnic differences. Seventh, multiple comparisons were not adjusted, increasing the risk of type I errors. Finally, we did not conduct analyses of clinical harm, cost, or cost-effectiveness, which are important for evaluating the practical implications of our screening protocol.

## Conclusion

In this research, rates of high-grade CIN and high-risk HPV infection were analysed across different regions, ethnicities, and age strata. Based on exploratory RF/SHAP modelling, our results suggested an association between age and the CIN2+ rate, and age, HPV16, and TCT were prominent exploratory predictors. Directly standardised comparisons did not support greater susceptibility by region, birthplace, or ethnicity, and genotype findings arose from incomplete site-dependent testing. However, given the limitations outlined above—including non-uniform extended genotyping, absence of confounder data, and sparse CIN2+ outcomes—these findings are hypothesis-generating and should be interpreted with caution. Moreover, our preliminary observations from the subset with extended genotyping suggest that HPV33, 52, and 58 may pose higher risks than other non-HPV16/18 types. But these findings require confirmation in future studies with uniform extended genotyping across all participants to fully validate their significance. Finally, while HPV genotyping and repeat positivity show potential for cancer risk estimation, independent validation, uniform genotyping, prespecified multivariable analysis, and health-economic evaluation are required before screening-interval or policy changes can be recommended.

## Data Availability

The participant-level data analyzed in this study are not publicly available due to privacy considerations. Requests for access should be directed to the corresponding author. Requests will be reviewed by the authors on a case-by-case basis in accordance with applicable ethics approvals and participant consent.
